# Suggestions and Comparisons of Two Algorithms for the Simplification of Bluetooth Sensor Data in Traffic Cordons

**DOI:** 10.3390/s24134375

**Published:** 2024-07-05

**Authors:** Beylun Özlü, Mustafa Sinan Yardım

**Affiliations:** 1 Transportation Ph.D. Program, Graduate School of Science and Engineering, Yildiz Technical University, Istanbul 34220, Turkey; 2 Civil Engineering Faculty, Civil Engineering Department, Yildiz Technical University, Istanbul 34220, Turkey; yardim@yildiz.edu.tr

**Keywords:** ITS, Bluetooth sensor, time series analysis, rule-based modeling, Istanbul historical peninsula, Jaccard similarity coefficient

## Abstract

Bluetooth sensors in intelligent transportation systems possess extensive coverage and access to a large number of identity (ID) data, but they cannot distinguish between vehicles and persons. This study aims to classify and differentiate raw data collected from Bluetooth sensors positioned between various origin–destination (i–j) points into vehicles and persons and to determine their distribution ratios. To reduce data noise, two different filtering algorithms are proposed. The first algorithm employs time series simplification based on Simple Moving Average (SMA) and threshold models, which are tools of statistical analysis. The second algorithm is rule-based, using speed data of Bluetooth devices derived from sensor data to provide a simplification algorithm. The study area was the Historic Peninsula Traffic Cord Region of Istanbul, utilizing data from 39 sensors in the region. As a result of time-based filtering, the ratio of person ID addresses for Bluetooth devices participating in circulation in the region was found to be 65.57% (397,799 person IDs), while the ratio of vehicle ID addresses was 34.43% (208,941 vehicle IDs). In contrast, the rule-based algorithm based on speed data found that the ratio of vehicle ID addresses was 35.82% (389,392 vehicle IDs), while the ratio of person ID addresses was 64.17% (217,348 person IDs). The Jaccard similarity coefficient was utilized to identify similarities in the data obtained from the applied filtering approaches, yielding a coefficient (J) of 0.628. The identity addresses of the vehicles common throughout the two date sets which are obtained represent the sampling size for traffic measurements.

## 1. Introduction

With the widespread adoption of Intelligent Transportation System (ITS) applications, Bluetooth sensors in particular have become commonly used advanced traffic measurement devices that can measure travel time, average speed, average progression and travel delays [1]. Bluetooth sensors also stand out due to factors such as accuracy, cost-effectiveness and ease of installation and use. With a coverage area of approximately 500 m, Bluetooth sensors do not differentiate between device types when identifying the MAC addresses of devices. A device with Bluetooth capability could belong to a vehicle’s Bluetooth device or various objects such as smartphones, smartwatches, computers, wireless headphones, micromobility vehicles or smart devices carried by passengers traveling in vehicles or public transportation. Bluetooth sensors detect the ID addresses of all objects with Bluetooth capability without filtering among them. Due to the widespread use of Bluetooth technologies, the variety of data coming from sensors has become a problem. When performing traffic calculations, it is not possible to produce realistic calculations in the current system by using the entire obtained data [2]. In a study conducted to evaluate the scope of Bluetooth technology, an experimental test environment was prepared, and it was indicated that the presence of multiple Bluetooth-containing objects could lead to a performance drop in the MAC protocol [3]. In later years, studies in the United States began to record and track data from Bluetooth sensors. By comparing the travel times generated by license plate reading devices on corridor lines with Bluetooth data, it was observed that average travel times yielded results that were 4–7% more accurate [4]. In a study conducted by Araghi et al. on the reliability of Bluetooth technology, it was found that a Bluetooth-enabled device would be detected with 80% reliability when passing through a sensor location [5]. Bluetooth, with its ability to provide real-time information as opposed to relying on past data, has been identified as a potential candidate for O-D estimation [6]. However, the literature indicates that continuous research is needed to maximize the potential of this technology in traffic management [7]. In Turkey, Bluetooth sensors are used for various purposes such as collecting travel data for different transportation modes, vehicle tracking and monitoring systems, fleet management for logistics companies, cargo tracking, monitoring urban parking areas and providing information about parking spaces. In a study conducted in Istanbul, a method was developed to produce average speed values for Istanbul’s urban traffic by using traffic measurement data obtained from sensors on highways, mobile application users and vehicle tracking systems [8]. Another study using air quality indicators and traffic index values for Istanbul included the changes in traffic index values and air quality indicators during the pandemic period [9]. In a study on intelligent mobility in Istanbul, both data obtained from traffic measurement sensors and vehicle tracking and mobile user data were processed through the Tukey Fences Clipping algorithm to bring outlier values in the data to normal values [10].

New approaches using machine learning techniques and tree-based algorithms through Bluetooth sensors are available to increase the reliability of Bluetooth-based traffic flow measurements and make it a more desirable and cost-effective solution for real-time traffic flow measurement [11]. In a study conducted to monitor real-time public transport passenger flow and O-D information based on Wi-Fi and Bluetooth sensors, a three-step, data-driven algorithm framework was proposed. The observed passenger flow was used as the ground truth to evaluate the performance of the proposed algorithm and according to the evaluation results, the proposed algorithm outperformed all selected baseline models and existing filtering methods [12]. In his study, Koçak indicated that determining travel time distribution using detectors with data fusion methods was successful, but Bluetooth sensor data were more reliable for OD matrices [13]. Bugdol conducted traffic prediction using standard Bluetooth data and obtained quite promising results [14]. In a study conducted on pedestrian traffic estimation with Bluetooth sensor technology, a detection methodology that included system calibration considering the likelihood of vehicle traffic congestion, travel time calculation and speed-based classification was proposed, and this methodology yielded an 89% accuracy rate in pedestrian detection [15].

The distribution of vehicles circulating in a region causes the planning of traffic routes. It is also used in determining alternative routes and planning public transportation. It allows traffic flow to be managed more efficiently, especially during peak hours. For these reasons, finding the distribution of data from sensors between vehicles and pedestrians allows traffic planning.

The purpose of this study is to simplify the data coming from Bluetooth sensors in cordon areas, such as city centers or busy commercial areas where transportation is heavy, to include only vehicles. For this purpose, two different simplification algorithms have been prepared. These algorithms include two basic filtering methods: “time-based filtering” and “speed-based filtering”. The results obtained from the Python code were compared with each other. The rule-based modeling used for speed-based filtering offers a simple and interpretable approach, but it may have some limitations in terms of flexibility and generalization power. Therefore, rule-based modeling is often compared with statistical methods and machine learning algorithms in the data analysis and modeling process. Statistical methods and machine learning aim to capture relationships and patterns in data in a more flexible and generalizable way. These methods allow for a broader understanding of the data and can be used to solve more complex problems. For this reason, a time-based filtering approach is also needed.

This article is structured as follows: Section 1 includes the definition of the problem and the review of similar studies in the literature. Section 2 provides an overview of the study’s methodology. Section 3 examines the Bluetooth sensor filtering algorithms, including their flowcharts, optimization procedures, and the model for comparing the two algorithms in detail. Section 4 presents the case study. It utilizes data from Bluetooth sensors positioned in the Historic Peninsula region of Istanbul, providing detailed information and addressing exceptional cases. Section 5 contains the results and discussion section, which includes a comparison of the computational efficiency of the two algorithms.

## 2. Methodology

**Figure 1:**
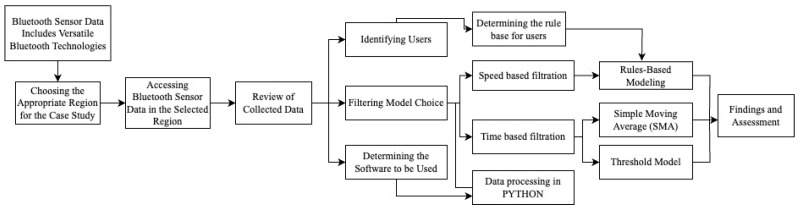
Flowchart.

As elements of intelligent transportation systems, Bluetooth sensors do not differentiate between device types when identifying the MAC addresses of devices and detect the ID addresses of all objects with Bluetooth capability without filtering among them. Due to the widespread use of Bluetooth technologies, the diversity of data coming from sensors has become a problem. It is necessary to simplify the data coming from Bluetooth sensors to include only vehicles. This study proposes two different filtering algorithms for simplification. These algorithms include two basic filtering methods: “time-based filtering” and “rule-based speed filtering”. The results obtained from the Python code were compared with each other. The comparison was made using the Jaccard similarity method. After the filtering was completed, cases where the identity addresses of devices matched, diverged, or were present in only one dataset were identified. The methodology of the study is presented below in the flowchart (Figure 1). Optimization procedures are detailed in Section 3.

## 3. Bluetooth Sensor Filtering Algorithm

### 3.1. Time-Based Filtering

Upon examining the accessed data, it is observed that a data series is formed over time and the data rows are arranged in a periodic cycle (s, h, days, months, years) [16]. The primary goal of analyzing and modeling time series data is to separate noise (the presence of random, meaningless, misleading data that complicate analysis and affect accuracy) from the dataset, thus obtaining an undisturbed series [17]. When filtering based on time, Simple Moving Average (SMA) and Threshold models, commonly used in time series analysis, are utilized. These two methods, which are statistical analysis tools, are frequently employed in data analysis processes. While SMA holds a significant place in time series analysis, the threshold model is commonly used in simple and fast decision-making processes. An algorithm utilizing these models was coded in the Python programming language and the dataset was parsed. At the end of the parsing, identity numbers (IDs) belonging to the designated categories of vehicle ID and person ID were obtained [2].

#### 3.1.1. Simple Moving Average (SMA)

If irregular movements dominate in a time series, a moving average model consisting of the following steps is used to determine the general trend of the series and reveal the cyclical effect [18]:

- Accumulation of data regarding the time series;
- Determination of the period;
- Calculation of the average;
- Updating calculations with each new data entry.

The moving average (SMA) value E(t) of the time during which the identity addresses of the devices are detected by the Bluetooth sensor is calculated using the equation below

(1) $$E(t)=1/N\;\sum_{i=0}^{N}\mathrm{tID}$$

- t_ID_: A series including the device ID detected more than once by the Bluetooth sensor;
- N: Number of periods in the average calculation.

When the Bluetooth sensors detect the ID identity address of the device, the time period during which the device is within the Bluetooth sensor coverage is recorded in a variable (t_BS_). t_bs_ represents the duration in which the device is within the Bluetooth sensor range.

#### 3.1.2. Threshold Model

This model, utilized to determine whether an event or condition will occur, typically sets a threshold and decides on further action based on whether the event surpasses this threshold. The following steps are followed in the Threshold Model:

1. Determining a threshold value according to the requirements of the research.
2. Utilizing the threshold value to make decisions based on data that fall above or below the threshold (Equation (2)).
3. Filtering based on these decisions.

Threshold Control:

t_BS_ > k · E(t) (2)

k: A coefficient that controls the relationship between the t_BS_ value and the threshold value (E(t)). If no specific coefficient is defined, the operation is performed for k = 1.

After calculating the total duration each device remained within the sensor coverage area for each device using the Group by function in PYTHON 3.11.9, E(t) is determined by taking the average of these durations. Finally, decisions are made for data above or below this threshold value. As it is known, in Equation (2), t_BS_ represents the time the Bluetooth device remains within the sensor range, while E(t) represents the weighted average of this time for all devices. If this condition in the equation is satisfied, it is assumed that the analyzed device belongs to the “person ID” address. Indeed, the threshold value here serves as an “acceptance function” in the person-vehicle separation in the study.

**Figure 2:**
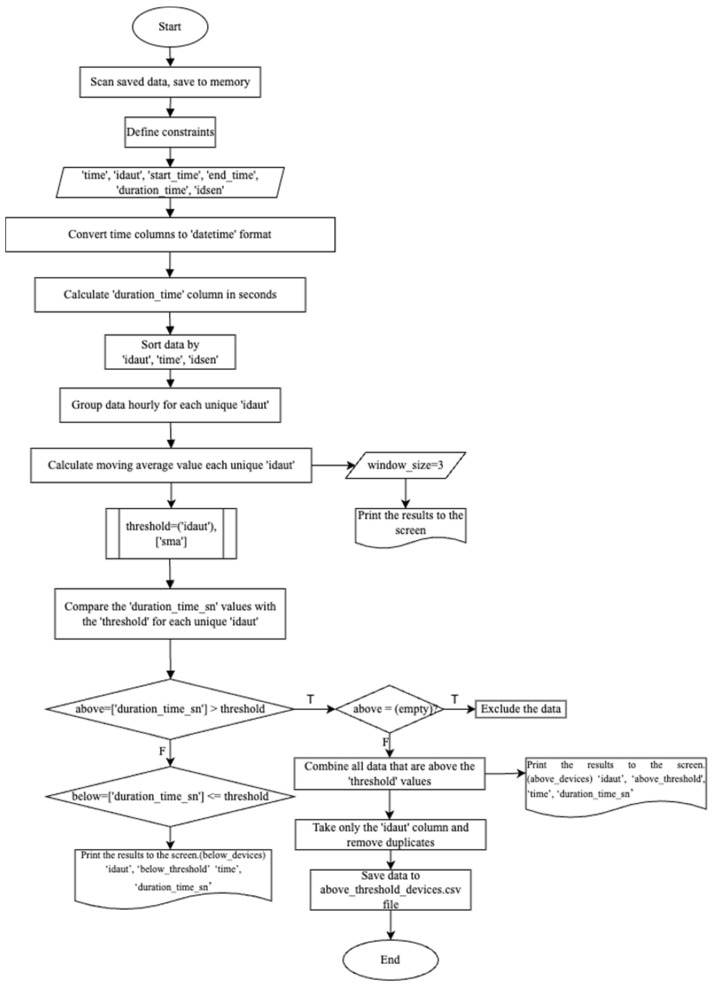
Bluetooth sensor time-based filtering algorithm.

The algorithm prepared to perform these operations in the Python programming language is presented in Figure 2.

The methodology using time series first calculates the Simple Moving Average (SMA). For this, data pertaining to the time series are collected, and a period is determined. Using code written in the PYTHON programming language, the average time each Bluetooth-equipped device spends at the sensor is determined. In the second part of the model, a threshold is used. In the threshold model, a threshold value is determined according to the research requirements. Here, the threshold value is the SMA (duration) value obtained for each device. Subsequently, using this threshold value, decisions are made, and filtering is performed based on whether the data are above or below the SMA. Accordingly, if a device spends more time at a sensor than its own average under non-traffic conditions, this device cannot belong to vehicles. This is because the time a moving device spends at a sensor under flowing traffic conditions is less compared to other conditions.

The value, called the threshold value and expressing the average time spent by Bluetooth devices between consecutive sensors, was examined for periodic time periods. As explained above, devices with remaining time values above the threshold value are in the ‘Personal Devices ID’ category and devices below are in the ‘Vehicle ID’ category.

**Figure 3:**
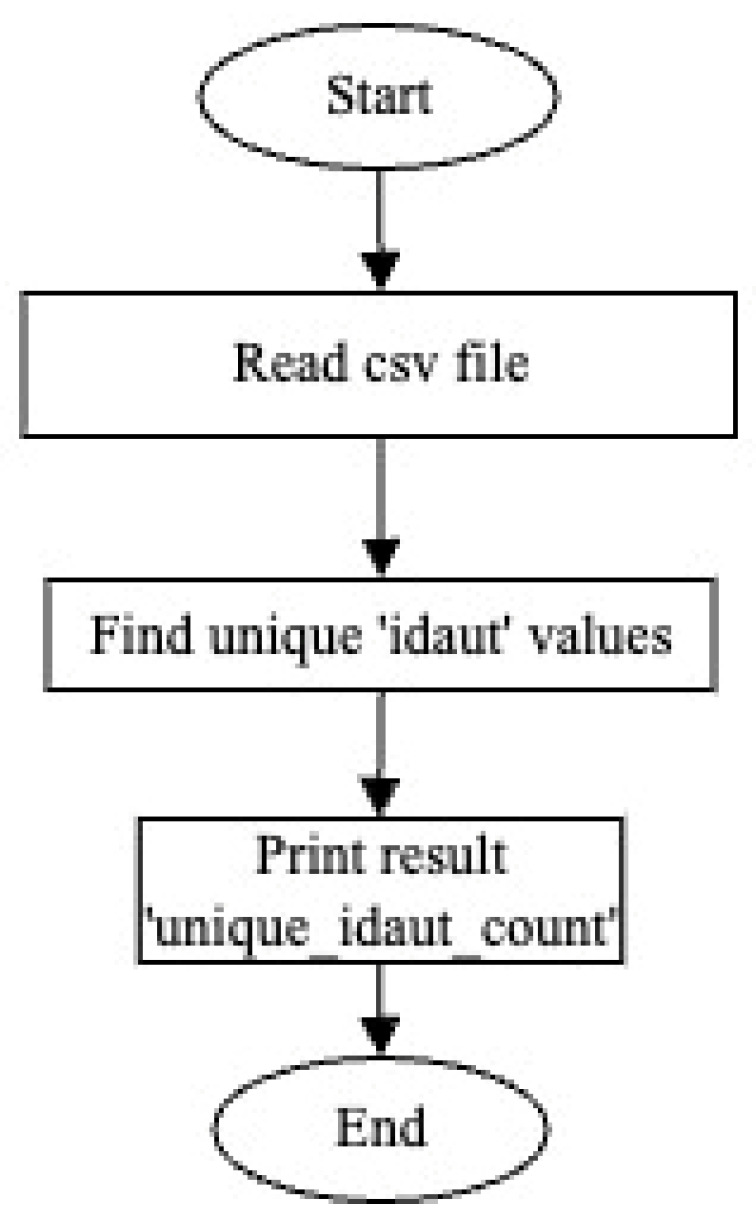
Finding the vehicle ID number as a result of filtering.

The above flow chart (Figure 2) was coded in Python programming language and the data were parsed; as a result, the matching Bluetooth devices were captured in the vehicle ID category (Figure 3). These data will be compared with the speed-based filtering algorithm in the next stage.

### 3.2. Speed-Based Filtering

This method is based on the rule-based modeling approach of speed values. This modeling can be explained in several stages:

- Obtaining distance data between sensors;
- Travel time calculation;
- Using two timestamps recorded by sensors to calculate trip travel time;
- Classification according to device speeds.

**Figure 4:**
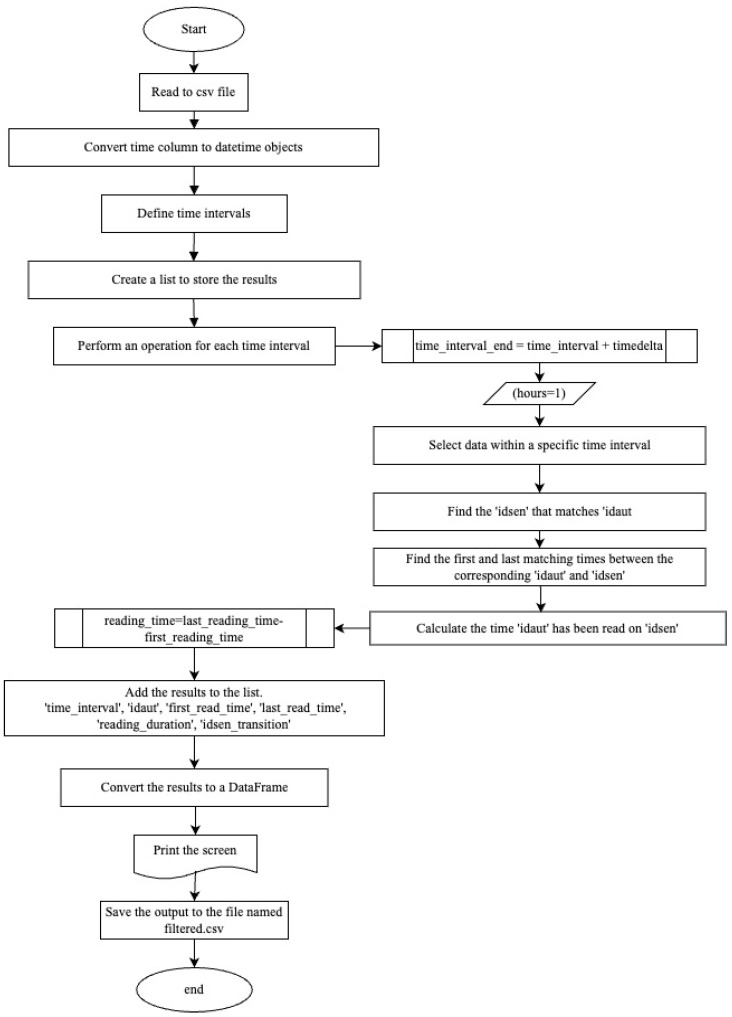
Bluetooth sensor data organizing.

For the filtering to be performed, speed data are required. To determine device speeds, both distance and time data are necessary. Firstly, for each device (idaut number), a code has been written to determine which sensor the device passed through in each 1 h time interval, the time it was first read by the Bluetooth sensor, the last time it was read and the duration it stayed at that sensor during the relevant time interval (in h:min). A code has been written for this purpose and the flow chart of the code is presented in Figure 4.

Then, speed measurements were made in 1 h periods for each device read from the Bluetooth sensors in the area. When calculating speed data, the following points must be considered:

- In cases where the device is read by only one sensor, the distance data are defined as 500 m since the sensor coverage area is 500 m.
- In cases where the device is read by more than one sensor, distance data are defined as the distance between sensors.
- In cases where the device’s residence time at the sensor was 00:00:00, no action was taken for this device. It was accepted that this device was not in the vehicle ID category.

**Figure 5:**
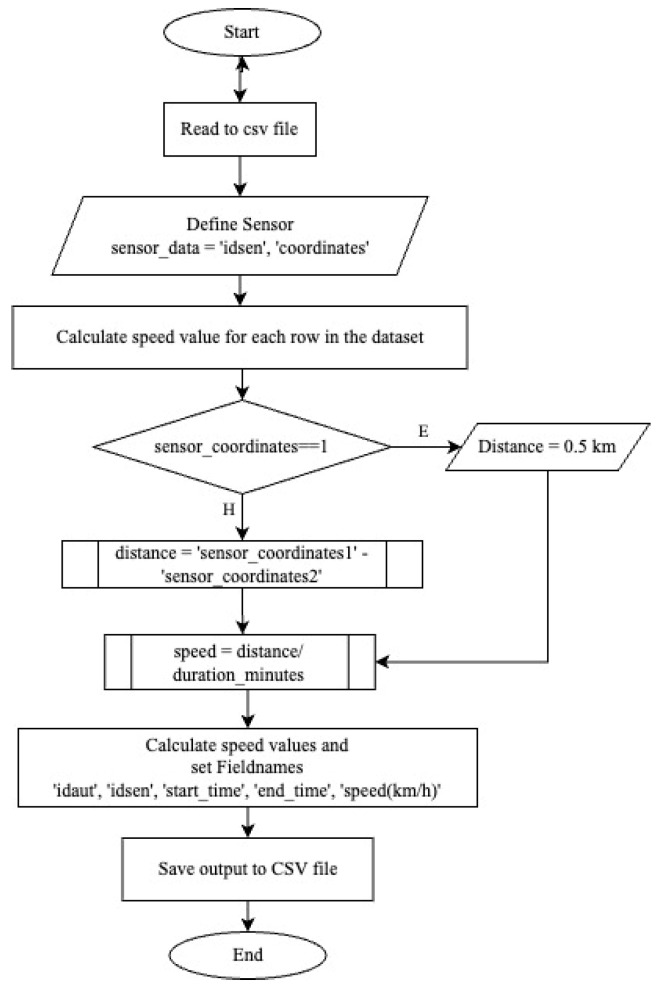
Bluetooth device speed calculation algorithm.

The relevant code and its output are presented in Figure 5.

The flow chart prepared to find the identification numbers of Bluetooth devices read by Bluetooth sensors and the passing speed of these devices through the sensors is as follows. The results will be discussed in the next section.

#### Rule-Based Modeling

Acceptances Based on Speed

- **Acceptance of Pedestrian (ID) Addresses**: Based on the extensive literature on pedestrian behavior in urban transportation and survey studies [19,20,21,22], an average pedestrian speed of 1.5 m/s (5.4 km/h) is chosen. Devices with speeds below 1.5 m/s are accepted as pedestrians.
- **Acceptance as Micromobility Vehicles:** The maximum speed of micromobility vehicles in Turkey is known to be 25 km/h. According to TomTom traffic data, the average congestion speeds in Istanbul during peak hours in 2023 were determined to be 27 km/h (morning) and 19 km/h (evening). Bicycle speeds range from 17 to 24 km/h, electric scooters from 19 to 24 km/h and electric motorcycles from 22 to 40 km/h [23]. In the selected region, assuming that devices with speeds between 5.4 km/h and 25 km/h belong to micromobility vehicles would disregard congestion conditions. Moreover, it is anticipated that Bluetooth devices used in micromobility vehicles in Istanbul transportation may not reflect the sample size. The traffic situation during peak and non-peak hours cannot serve as a basis for distinguishing between transportation modes. Therefore, speeds between 5.4 km/h and 25 km/h will not be used as micromobility speeds.
- **Acceptance of Users Traveling on Public Transport:** In the region, where the maximum number of lanes is 8, if more than 8 ID numbers traveling at the same speed during the same time interval are detected, they are assumed to belong to users traveling on public transport.
- **Acceptance of Pedestrians Traveling Together:** If devices with the same ID number are matched within the same sensors during the same time interval and have the same speed and if the speed is below 1.5 m/s (5.4 km/h), it is assumed that these devices belong to users traveling together as pedestrians.
- **Acceptance of Bluetooth Devices Belonging to Residents:** This is an acceptance used for devices with a speed value of 0 km/h, indicating that they cannot participate in circulation within the region for various reasons but have been detected multiple times by the sensors. These devices do not fall under the vehicle ID category.
- **Acceptance of Vehicle Identification (ID) Addresses:** Devices with speeds above 1.5 m/s (5.4 km/h) and determined to be traveling singularly belong to the vehicle category.

**Figure 6:**
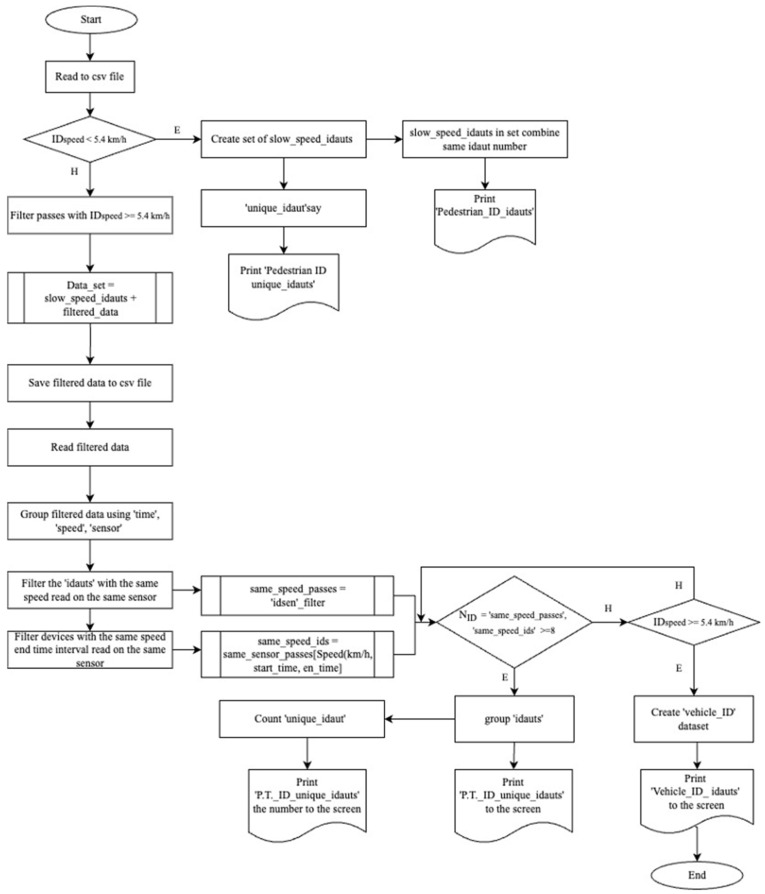
Rule-based modeling algorithm according to speed values.

At the end of this classification, device identification numbers belonging to different categories were identified. We can see the classification algorithm and the time spent by vehicle identification addresses and the method of finding their speed in the flow chart below. This flow chart (Figure 6) is converted into code in the Python 3.11.9 software language and vehicle and pedestrian distribution is achieved.

### 3.3. Jaccard Similarity Coefficient

The data accessed as a result of the applied filtering approaches appear to be close to each other in terms of numerical values. However, what is obtained in the result set are two different datasets containing ID numbers of devices with Bluetooth connection. It would not be a realistic approach to expect the IDs in both datasets to match each other exactly. Therefore, the similarities of the models need to be determined.

**Figure 7:**
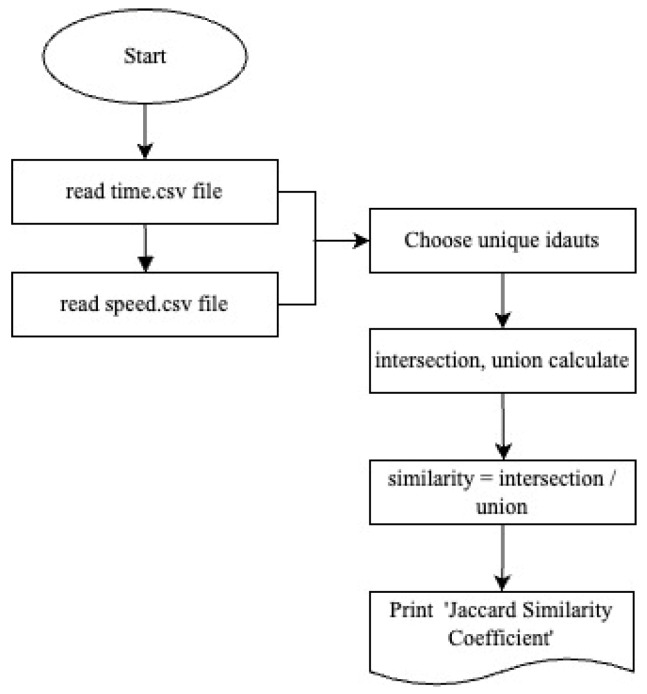
Jaccard similarity coefficient calculation diagram.

The Jaccard index, also known as the Jaccard similarity coefficient, is a widely used measure of similarity between sets of models in various fields such as data mining, clustering and genomics [23]. Overall, the Jaccard index stands out as a fundamental similarity measure with wide applications in various disciplines and offers a standardized approach to measuring similarity between datasets (Figure 7).

The Jaccard index measures the similarity between two clusters by calculating the ratio of the number of elements common to both clusters to the total number of different elements in the clusters [24]. The resulting value varies between 0 and 1; 0 represents no overlap between clusters, and 1 represents complete overlap [25]. Two different datasets containing ID numbers of devices with Bluetooth connection accessed as a result of the applied filtering approaches were run in the code written to detect the Jaccard index.

**Figure 8:**
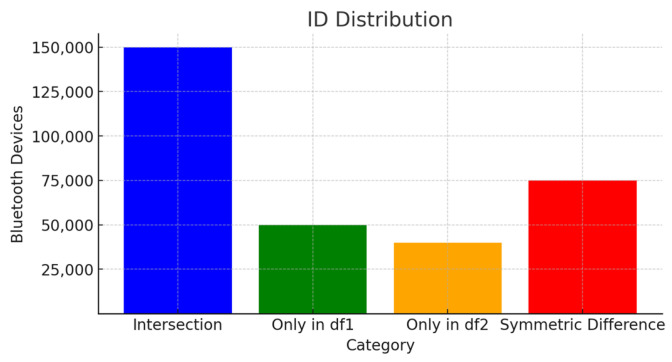
Bluetooth devices distribution for Jaccard similarity coefficient.

In the graph in the figure (Figure 8), vehicle identities that intersect with each other in the datasets consisting of Bluetooth identities of the vehicles (Intersection), vehicle identities that are only in the cluster formed as a result of the time-based algorithm (only in df1), vehicle identities that are only in the cluster resulting from the speed-based algorithm (only in df2) and discrete distributions of vehicle identities (Symmetric Difference) of the elements are shown.

## 4. Case Study

### 4.1. Selection of the Study Area

The Historic Peninsula of Istanbul, where many transportation modes are used together, serves as a transit route for Istanbul, and data collected from Bluetooth sensors play an important role in intelligent transportation system-based planning. Additionally, due to data availability, the Historic Peninsula of Istanbul is considered a suitable example area. Specific points that can serve as start and end points have been designated in this area, known as the cordon area, and data from 39 Bluetooth sensor devices located between the I–J point pairs have been used.

### 4.2. Access to Bluetooth Sensor Data

In the application part of the study, data from sensors positioned at various locations near the entry and exit points (gates) of the cordon area were accessed through relevant official organizations. The accessed data include:

- Bluetooth sensor numbers (idsen);
- Identity (ID) addresses of objects with Bluetooth devices (idaut);
- Time information at the moment the Bluetooth sensor and device pairing is established (in the format Day-Month-Year; h-min-s) (time).

### 4.3. User Identification

Service users within the coverage area of the Bluetooth sensor in the study area were identified and classified based on information provided by official institutions and observation-based field studies as follows:

- Identity (ID) addresses of vehicles (Private Vehicles, Heavy Vehicles, Light Commercial Vehicles, Ships, Public Transport Vehicles);
- Identity (ID) addresses of individuals (Smartphones, smartwatches, wireless headphones belonging to pedestrians, Bluetooth devices carried by passengers traveling on public transport or in vehicles, bicycles, motorcycles, pedestrians traveling on micromobility vehicles, Bluetooth devices carried by multiple pedestrians traveling together).

### 4.4. Bluetooth Sensor Location Review

**Figure 9:**
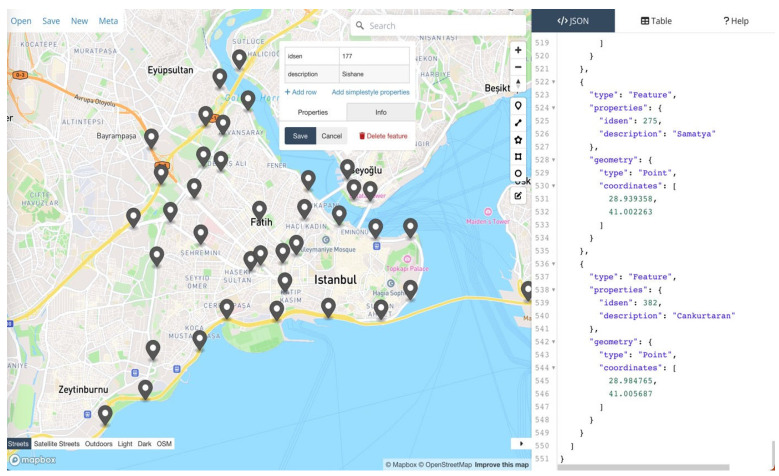
Bluetooth sensor devices view in coordinates.

Bluetooth sensors in their positioned state within the area are shown in Figure 9.

The distribution of Bluetooth sensors in the İstanbul Peninsula Historical area, along with their sensor numbers, is shown in Table 1.

### 4.5. Bluetooth Sensor Data

Data from the period between 1 April 2023 and 16 April 2023 were accessed. During this time, a dataset with 8,282,609 rows was examined in Python 3.11.9.

Out of the 39 sensors shown in the location information, data from 35 sensors are present in the dataset. The sensor numbers that appear in the location information but are not in the dataset are 73, 96, 228 and 220. At sensor number 62, data were only read once on 4 April 2023, at 11:43 a.m.

**Figure 10:**
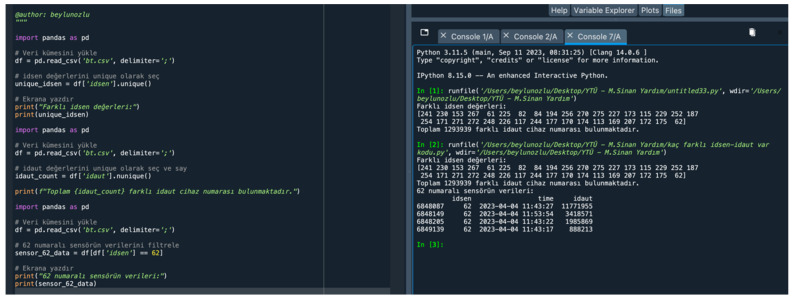
Sensor information.

Within these data, a total of 1,293,939 different Bluetooth sensor devices read by 35 different sensor numbers (idsen) (241, 230, 153, 267, 61, 225, 82, 84, 194, 256, 270, 275, 227, 173, 115, 229, 252, 187, 254, 171, 271, 272, 248, 226, 117, 244, 177, 170, 174, 113, 169, 207, 172, 175, 62) were identified. The code used for this process and the output of the code are as follows (Figure 10).

### 4.6. Distance Matrix between Bluetooth Sensors

**Figure 11:**
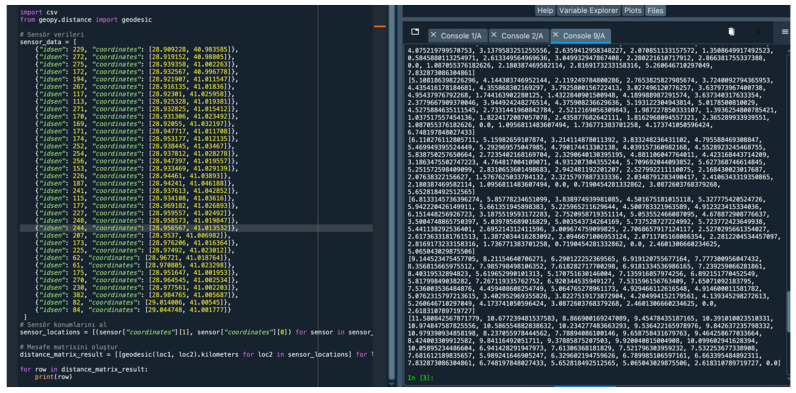
Bluetooth sensor distance matrix code and output.

While creating the distance matrix for Bluetooth sensor locations, the Python–Spyder Geopy library was loaded and the geojson file was displayed in the Python script. The Geopy. distance module, which contains the Geodesic function required for the correct use of the Geopy library and distance calculations, was used. The code (Figure 11) written for the distance matrix obtained from the location data is as follows.

The distance matrix obtained from the location data is as follows (Table 2).

### 4.7. Bluetooth Sensor Data Examination

In the Bluetooth sensor data sorted by time, the first column named idsen represents the sensor number, the second column named idaut represents the identity number of the Bluetooth device that passed by the sensor and the third column named time shows the time when the device was read by the Bluetooth sensor.

The Bluetooth sensors have a horizontal coverage of 110° and a vertical coverage of 30°, with a range of slightly over 500 m (according to information from the manufacturer). Data are transmitted from the Bluetooth sensors to the center in 3 min intervals.

Bluetooth sensor data from the period between 1 April 2023 and 16 April 2023 were accessed. During this time, a dataset of 8,282,609 rows was examined in Python.

When examining the data, device identities (ID) (idaut) that were recorded only once in the dataset by 35 sensors between 1 April 2023 and 16 April 2023 were identified.

#### Bluetooth Devices Detected Just Once in the Dataset

Considering the coverage area of the Bluetooth sensors and the time in which data are transmitted to the center:

- **Being in the Personal Devices ID Category:** It is known that data are transmitted to the center every 3 min. If the device belongs to the person ID category, it cannot be read just once within 3 min. Even when the speed value is 0 km/h, it is known that the sensor would read the device multiple times while it is within the coverage area. Therefore, the device ID could have been read due to the Bluetooth device being turned on and off within the region. In this case, since there is no second read time for the ID, no speed data can be accessed.
- **Being in the Vehicle ID Category:** Device IDs read just once in the region could only have been identified as belonging to a vehicle moving along the periphery of the cordon area, considering the sensor coverage area. In this case, it would be meaningless to include them in the pilot area traffic counts.

**Figure 12:**
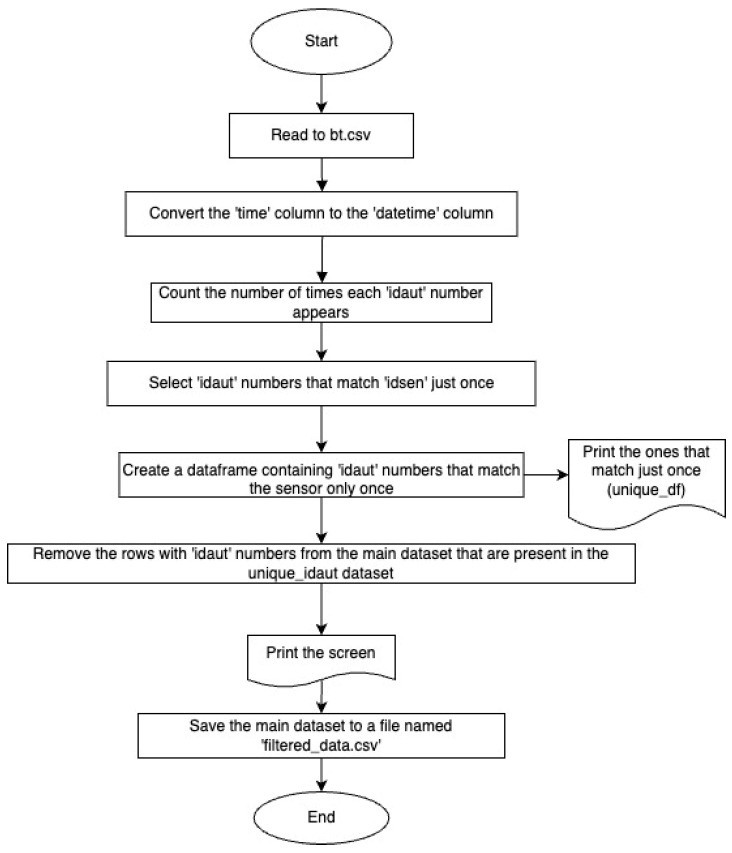
Identification of Bluetooth devices read just once and their removal from the main dataset.

As a result, excluding Bluetooth device identity numbers (IDs) that were read just once in the entire dataset from the main dataset is a necessary step to form the sample size. The flowchart (Figure 12) created to perform this process in the Python programming language is as follows.

**Figure 13:**
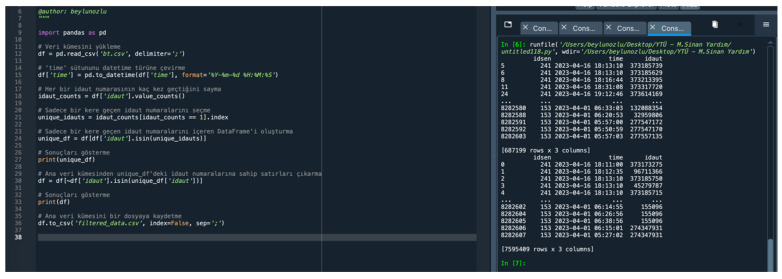
Identification and removal of Bluetooth devices read just once from the main dataset.

There are a total of 687,199 identity numbers of Bluetooth devices that were read only once during the 16 days from 1 April 2023 to 16 April 2023 in the main dataset. When the devices read only once are removed from the main dataset, there are 606,740 identity numbers of Bluetooth devices remaining and the dataset consists of 7,595,410 rows (Figure 13). Future studies will be conducted for these devices.

## 5. Results and Discussion

### 5.1. Bluetooth Sensor Speed-Based Filtering Algorithm Results

To determine device speeds, both distance and time data are necessary. A code (Figure 4) was developed to track each device (identified by an idaut number) through the sensors. This code records which sensor the device passed through within each 1 h time interval, the first and last time the Bluetooth sensor read the device, and the duration the device remained at each sensor (in h). The output of this code is presented in Table 3.

A section from the table showing the identification numbers of Bluetooth devices read by Bluetooth sensors and the passing speed of these devices through the sensors between 1 April 2023 and 16 April 2023 is presented below (Table 4). These results were obtained by running the flow chart in Figure 5 (Bluetooth Device Speed Calculation Algorithm) in Python.

According to the acceptances made in the section Acceptances Based on Speed (section Rule-Based Modeling), speeds of devices for hourly periods from 1 April 2023 to 16 April 2023, were classified. At the end of this classification, device identification numbers belonging to different categories were identified. This classification was made with the help of the flow chart shown in Figure 6 (Rule-Based Modeling Algorithm According to Speed Values). A snippet from the table (Table 5) showing the time spent by vehicle ID addresses and their speeds is provided below.

### 5.2. Bluetooth Sensor Time-Based Filtering Algorithm Results

By converting the flow chart in Figure 7 into code in Python, the value, called the threshold value, which expresses the average time spent by Bluetooth devices between consecutive sensors, was examined for periodic time periods. Devices with remaining time values above the threshold value are in the ‘Personal Devices ID’ category and devices below are in the ‘Vehicle ID’ category.

By coding Figure 8 (Finding the vehicle ID number as a result of filtering) flowchart in Python programming language, the data were parsed; as a result, 208,941 different Bluetooth device matches were captured in the vehicle ID category. The number of paired Bluetooth devices belonging to individuals was found to be 397,799. According to this filtering method using time series, 34.43% of Bluetooth devices are in the vehicle ID category, while 65.57% are in the person ID category. These data will be compared with the filtering algorithm based on speed in the next stage.

### 5.3. Comparison of the Modelling Results

In this study, two different filtering algorithms are considered. The first of these is the analysis made using time series. As a result of this analysis, for Bluetooth devices circulating in the region, if Equation (2) is correct, the rate of ID addresses belonging to people is 65.57% (397,799 personal devices ID), while the rate of ID addresses belonging to vehicles is 34.43% (208,941 vehicle ID) has happened. Another model designed is rule-based modeling based on speed. This model establishes an algorithm for determining the speed data of devices and then makes classifications for the speed data obtained. In light of these data, the rate of identity (ID) addresses of individuals was found to be 64.17% (217,348 personal devices ID), while the rate of identity (ID) addresses of vehicles was found to be 35.82% (389,392 vehicle ID). Here, the personal devices ID set includes devices with Bluetooth found on objects owned by pedestrians (pedestrian ID), device IDs with Bluetooth found on objects owned by people traveling in public transportation (P.T. ID) and residents or people in the region who cannot participate in the calculation because the speed value is 0. It is distributed into different subsets such as Bluetooth devices (L.R. ID) belonging to objects. The results are seen in Table 6 and Table 7.

The data accessed as a result of the applied filtering approaches appear to be close to each other in terms of numerical values. However, what is obtained in the result set are two different datasets containing ID numbers of devices with Bluetooth connection. It would not be a realistic approach to expect the IDs in both datasets to match each other exactly. Therefore, the similarities of the models need to be determined. The Jaccard index stands out as a fundamental similarity measure with wide applications in various disciplines and offers a standardized approach to measuring similarity between datasets. The Jaccard index value was calculated between two datasets found as a result of the algorithms.

The Jaccard index value (J) calculated as a result of the transactions was found to be 0.628. Considering the index value varying between 0 and 1, it can be seen that the overlap between the data is at a satisfactory level. This method can be considered as a parameter that will indirectly show the success rate of the models.

### 5.4. Discussion and Suggestions

In the literature, studies utilizing Bluetooth sensors for traffic measurements have primarily focused on the issue of sensor placement [26,27,28], striving to obtain accurate data rather than merely segregating the data. However, with the advancement of Bluetooth technologies, this approach has become increasingly challenging. To enhance the reliability of Bluetooth-based traffic measurements and make real-time traffic flow measurement more desirable and cost-effective, new approaches have been introduced, utilizing machine learning techniques and tree-based algorithms with Bluetooth sensors [11]. In a study where modes such as walking, cycling, tram, bus, taxi, and private vehicle were categorized based on data collected via GPS from smartphones, significant features from mobility models, including speed, acceleration, and rapid acceleration, were extracted and longitudinal dynamics were applied to train the classification model. During the model validation for the classification algorithm, a decision tree that achieved high accuracy in testing was employed [29]. Although our study, which involves classifying transportation modes using a classification algorithm, demonstrates performance similar to the machine learning approaches in Study [29], it differs in comparison due to the type of data and the complexity of the statistical model used. It is anticipated that with the integration of other systems, given known distributions, it will be critically important for real-time traffic monitoring applications in the future.

The biggest problem encountered while conducting the study was the inability to access data from some Bluetooth sensors or the presence of incomplete data in the case study area, due to maintenance and repair conditions. This situation, which arose due to the nature of existing Bluetooth sensors, made working with the current sensors challenging. Therefore, sensors that did not contain missing or faulty data were used within the planned timeframe.

While concentrating on the Historic Peninsula of Istanbul enables a comprehensive understanding of the city’s traffic congestion dynamics, unique factors such as population density, infrastructure layout, and cultural influences may constrain the direct applicability of our results to larger or differently structured cities. Future research should address these disparities to enhance the transferability of our findings to diverse urban environments.

### 5.5. Suggestions

The issue of determining the location for Bluetooth sensors is another problem that needs to be addressed. Specifically, placing Bluetooth sensors in the correct locations (preferably with high vehicle traffic and low pedestrian traffic) is a step towards solving this complexity, particularly for urban traffic measurements.

With the proliferation of smart devices, the number of Bluetooth-equipped devices is increasing daily. This trend cannot be prevented. However, it is proposed to add a data-differentiating parameter for vehicle Bluetooth in the dataset; this proposal can only be implemented systemically in new-generation vehicles.

Algorithms based on statistical methods and rule-based modeling designed in the study make it easier to parse data from Bluetooth sensors. It is important to know which of the ID device identification numbers that match Bluetooth sensors belong to the vehicles to use these devices in transportation systems. The algorithms used make it easier to work with data by reducing noise in the data, but it would be useful to support Bluetooth technology with other systems at certain points to verify the data and calibrate the results.

## 6. Concluding Remarks and Future Perspectives

This paper proposes two innovative algorithms for filtering Bluetooth sensor data. The first algorithm involves a simplification process using time series based on the Simple Moving Average (SMA) and threshold models, which are tools of statistical analysis. The second algorithm employs a rule-based simplification mechanism where the data obtained from the sensors are processed and the location information of the sensors is incorporated, utilizing the speed values of the Bluetooth devices accessed. The variables of the filtering problem include the times at which Bluetooth-enabled devices matched with the sensors at specific intervals, the durations for which these devices initially and finally matched with the sensors, the distances between the sensors, the durations the devices stayed within the sensors’ range, and the speed values of these devices. The application of the proposed algorithms resulted in a close similarity in the proportions of identified vehicle identity addresses (ID) (time-based filtering algorithm 34.43%; speed-based filtering algorithm 35.82%). Device identities that are common in the dataset obtained as a result of the algorithms are expressed as an intersection set. The similarity between the results of the two algorithms was calculated using the Jaccard similarity index (JI: 0.628). The statistical methods and rule-based models developed in this study significantly facilitate the segregation of data obtained from Bluetooth sensors. Identifying which device identity numbers matched with Bluetooth sensors belong to vehicles is crucial for utilizing these devices in transportation calculations.

In the subsequent phase of the research, origin-destination (O-D) matrices will be created to enable traffic planning and measurements using the obtained vehicle identity devices (vehicle ID). The filtering conducted allows for the creation of a distribution matrix when the Bluetooth sensor locations in the studied area are taken as the origin and destination points. The sequential data from Bluetooth sensors allow for tracking the routes of vehicles by observing which sensors they pass through and in what order. In future studies, the distribution ratios of devices matched by sensors in a specific region can be determined. The distribution matrix serves as a fundamental tool for any operations to be conducted in the desired region. Knowing the distribution of vehicles or persons in the area and placing them within the matrix provides decision-makers, especially in cordon-type enclosed areas, with a sustainable decision-making tool for short- and long-term regional transportation decisions. Also, knowing these distribution ratios will enable the achievement of real-time data flow by supporting it with other systems. In future studies, the separation of vehicles and persons will allow for the determination of which areas are more exposed to air pollutants. Furthermore, based on measurement and distribution matrices, sustainability assessments for air pollutants in urban transportation are planned.

## Figures and Tables

**Table 1:** Distribution of Bluetooth sensors in the historical peninsula region.

Gates	Name	Bluetooth Sensor No (idsen)
Yedikule	Yedikule Coastel	229
	Yedikule	272
	SamaTya	275
	Samatya Coastel	172
	10.Yil Avenue	73
	10.Yil Avenue 2	194
	Haseki	96
Topkapı	Topkapi	267
	Topkapi Cemetery	117
	Pazartekke	113
	Capa	271
Ulubatlı	Vatan Avenue	170
	Bayrampasa	169
	Ahmediye	171
	Sarachane	174
Edirnekapi	Edirnekapi	252
	Karagumruk	254
	Fatih Mosque	256
	Edirnekapi-1	153
Ayvansaray	Balat Hospital	226
	Halicioglu	187
	Feshane	241
	D100 Otakcilar	115
Unkapani	Sishane	177
	Unkapani Coastel	227
	IMC	248
	Istanbul Municipality	244
	Kucuk Langa	207
Galata Bridge	Sarayburnu Coastel	228
	Sirkeci	173
	Karakoy Square	225
	İstanbul Ticaret University	62
	Karakoy	61
Avrasya Tunnel	Yenikapı Coastel	175
	Kumkapi	270
	Kumkapi Coastel	230
	Cankurtaran	282
	Harem	82
	Acibadem	84

**Table 2:** Distance matrix between Bluetooth sensors.

OD		Yedikule Sahil							Topkapı				Ulubatlı				Edirnekapı				Ayvansaray				Unkapanı					Galata Köprüsü					Avrasya Tüneli					
	BS No	229	272	275	172	73	194	96	267	117	113	271	170	169	171	174	252	254	256	153	226	187	241	115	177	227	248	244	207	228	173	225	62	61	175	270	230	282	82	84
Yedikule Sahil	229	0	0.97	3.28	2.45	1.61	3.28	4.27	3.91	4.85	4.2	4.05	4.81	5.48	4.5	4.87	6.18	5.52	5.13	5.45	6.83	7.49	7	6.2	6.97	6.25	5.78	5.19	4.56	7.34	6.71	7.05	6.25	6.8	4.11	5.11	6.11	6.81	9.14	11.58
	272	0.97	0	2.32	1.49	0.8	2.62	3.34	3.38	4.22	3.52	3.25	4.07	4.9	3.56	3.92	5.43	4.74	4.23	4.72	6.04	6.75	6.28	5.49	6.03	5.32	4.84	4.23	3.59	6.37	5.74	6.09	5.29	5.85	3.14	4.14	5.16	5.86	8.21	10.68
	275	3.28	2.32	0	0.84	1.74	1.79	1.06	2.65	2.97	2.24	1.56	2.45	3.68	1.26	1.6	3.6	2.89	2.04	3.03	4.1	4.88	4.51	3.79	3.71	3.04	2.53	1.91	1.32	4.14	3.47	3.78	2.97	3.53	1.03	2.12	3.21	3.84	6.29	8.87
	172	2.45	1.49	0.84	0	0.99	1.87	1.88	2.77	3.34	2.58	2.07	2.97	4.06	2.09	2.43	4.24	3.53	2.82	3.59	4.79	5.55	5.13	4.38	4.55	3.86	3.37	2.75	2.11	4.92	4.27	4.6	3.8	4.36	1.71	2.77	3.83	4.5	6.92	9.45
	73	1.61	0.8	1.74	0.99	0	1.83	2.68	2.62	3.43	2.72	2.47	3.27	4.12	2.91	3.31	4.64	3.95	3.51	3.93	5.26	5.96	5.49	4.69	5.38	4.64	4.19	3.63	3.06	5.88	5.21	5.5	4.7	5.23	2.69	3.76	4.83	5.5	7.91	10.44
	194	3.28	2.62	1.79	1.87	1.83	0	1.96	0.9	1.6	0.92	1.01	1.54	2.3	2.17	2.63	2.92	2.29	2.32	2.18	3.59	4.22	3.72	2.92	4.33	3.5	3.22	2.92	2.72	5.32	4.6	4.64	3.89	4.32	2.72	3.72	4.8	5.33	7.78	10.39
	96	4.27	3.34	1.06	1.88	2.68	1.96	0	2.59	2.52	1.93	1.17	1.84	3.16	0.24	0.69	2.72	2.05	1	2.27	3.13	3.95	3.63	2.98	2.7	1.98	1.51	1	0.82	3.4	2.68	2.85	2.05	2.56	1.11	1.86	2.88	3.37	5.81	8.43
Topkapı	267	3.91	3.38	2.65	2.77	2.62	0.9	2.59	0	1.02	0.78	1.44	1.4	1.58	2.76	3.19	2.61	2.13	2.63	1.89	3.31	3.8	3.27	2.49	4.56	3.72	3.57	3.44	3.4	5.78	5.06	4.97	4.3	4.63	3.5	4.44	5.47	5.94	8.36	10.97
	117	4.85	4.22	2.97	3.34	3.43	1.6	2.52	1.02	0	0.76	1.43	0.75	0.72	2.61	2.97	1.62	1.27	2.17	0.95	2.32	2.78	2.24	1.47	3.88	3.08	3.07	3.14	3.33	5.31	4.6	4.38	3.8	4.03	3.59	4.36	5.29	5.66	7.99	10.59
	113	4.2	3.52	2.24	2.58	2.72	0.92	1.93	0.78	0.76	0	0.77	0.68	1.48	2.07	2.48	2.02	1.44	1.86	1.28	2.71	3.31	2.8	2	3.78	2.94	2.8	2.71	2.76	5.02	4.29	4.19	3.52	3.85	2.94	3.79	4.79	5.23	7.62	10.23
	271	4.05	3.25	1.56	2.07	2.47	1.01	1.17	1.44	1.43	0.77	0	0.91	2.13	1.32	1.75	2.19	1.49	1.31	1.53	2.79	3.51	3.07	2.31	3.31	2.48	2.22	2.01	1.99	4.38	3.65	3.64	2.92	3.31	2.18	3.03	4.04	4.5	6.92	9.54
Ulubatlı	170	4.81	4.07	2.45	2.97	3.27	1.54	1.84	1.4	0.75	0.68	0.91	0	1.32	1.9	2.23	1.38	0.76	1.42	0.65	2.05	2.69	2.21	1.43	3.21	2.38	2.33	2.4	2.63	4.57	3.86	3.67	3.07	3.32	2.94	3.64	4.55	4.91	7.24	9.84
	169	5.48	4.9	3.68	4.06	4.12	2.3	3.16	1.58	0.72	1.48	2.13	1.32	0	3.22	3.53	1.53	1.52	2.66	1.14	2.16	2.41	1.86	1.22	4.13	3.38	3.48	3.67	3.95	5.68	5	4.68	4.2	4.34	4.26	4.95	5.84	6.15	8.4	10.98
	171	4.5	3.56	1.26	2.09	2.91	2.17	0.24	2.76	2.61	2.07	1.32	1.9	3.22	0	0.46	2.67	2.02	0.87	2.28	3.03	3.86	3.56	2.95	2.47	1.77	1.28	0.77	0.73	3.17	2.45	2.61	1.82	2.33	1.13	1.74	2.72	3.19	5.62	8.24
	174	4.87	3.92	1.6	2.43	3.31	2.63	0.69	3.19	2.97	2.48	1.75	2.23	3.53	0.46	0	2.79	2.21	0.96	2.51	3.06	3.89	3.65	3.11	2.12	1.52	0.97	0.32	0.57	2.71	1.99	2.19	1.39	1.93	1.14	1.43	2.33	2.75	5.17	7.79
Edirnekapı	252	6.18	5.43	3.6	4.24	4.64	2.92	2.72	2.61	1.62	2.02	2.19	1.38	1.53	2.67	2.79	0	0.71	1.84	0.74	0.7	1.32	0.91	0.4	2.73	2.08	2.36	2.8	3.33	4.39	3.77	3.33	3	3	3.8	4.19	4.88	5.05	7.14	9.66
	254	5.52	4.74	2.89	3.53	3.95	2.29	2.05	2.13	1.27	1.44	1.49	0.76	1.52	2.02	2.21	0.71	0	1.26	0.38	1.31	2.03	1.62	0.93	2.64	1.87	1.98	2.27	2.72	4.17	3.49	3.18	2.69	2.83	3.15	3.64	4.42	4.68	6.89	9.46
	256	5.13	4.23	2.04	2.82	3.51	2.32	1	2.63	2.17	1.86	1.31	1.42	2.66	0.87	0.96	1.84	1.26	0	1.58	2.16	2.99	2.71	2.16	2.01	1.18	0.94	1.02	1.49	3.17	2.45	2.35	1.67	2.01	1.99	2.38	3.19	3.5	5.82	8.42
	153	5.45	4.72	3.03	3.59	3.93	2.18	2.27	1.89	0.95	1.28	1.53	0.65	1.14	2.28	2.51	0.74	0.38	1.58	0	1.44	2.04	1.56	0.78	3.01	2.24	2.35	2.6	2.99	4.54	3.86	3.55	3.06	3.21	3.38	3.94	4.76	5.04	7.27	9.84
Ayvansaray	226	6.83	6.04	4.1	4.79	5.26	3.59	3.13	3.31	2.32	2.71	2.79	2.05	2.16	3.03	3.06	0.7	1.31	2.16	1.44	0	0.83	0.73	0.94	2.46	2	2.42	2.99	3.63	4.2	3.65	3.1	2.94	2.8	4.15	4.38	4.93	5	6.92	9.38
	187	7.49	6.75	4.88	5.55	5.96	4.22	3.95	3.8	2.78	3.31	3.51	2.69	2.41	3.86	3.89	1.32	2.03	2.99	2.04	0.83	0	0.55	1.31	3.11	2.77	3.23	3.82	4.46	4.86	4.36	3.75	3.69	3.49	4.97	5.19	5.71	5.74	7.53	9.92
	241	7	6.28	4.51	5.13	5.49	3.72	3.63	3.27	2.24	2.8	3.07	2.21	1.86	3.56	3.65	0.91	1.62	2.71	1.56	0.73	0.55	0	0.8	3.19	2.71	3.1	3.63	4.21	4.93	4.38	3.83	3.65	3.54	4.69	5.02	5.63	5.72	7.65	10.1
	115	6.2	5.49	3.79	4.38	4.69	2.92	2.98	2.49	1.47	2	2.31	1.43	1.22	2.95	3.11	0.4	0.93	2.16	0.78	0.94	1.31	0.8	0	3.12	2.48	2.74	3.14	3.64	4.79	4.17	3.73	3.39	3.4	4.08	4.53	5.25	5.44	7.54	10.06
Unkapanı	177	6.97	6.03	3.71	4.55	5.38	4.33	2.7	4.56	3.88	3.78	3.31	3.21	4.13	2.47	2.12	2.73	2.64	2.01	3.01	2.46	3.11	3.19	3.12	0	0.84	1.19	1.82	2.57	1.75	1.31	0.65	0.92	0.42	3.14	2.73	2.83	2.7	4.46	6.94
	227	6.25	5.32	3.04	3.86	4.64	3.5	1.98	3.72	3.08	2.94	2.48	2.38	3.38	1.77	1.52	2.08	1.87	1.18	2.24	2	2.77	2.71	2.48	0.84	0	0.57	1.29	2.05	2.32	1.69	1.31	0.94	0.96	2.64	2.52	2.94	3.01	5.06	7.61
	248	5.78	4.84	2.53	3.37	4.19	3.22	1.51	3.57	3.07	2.8	2.22	2.33	3.48	1.28	0.97	2.36	1.98	0.94	2.35	2.42	3.23	3.1	2.74	1.19	0.57	0	0.72	1.49	2.24	1.53	1.42	0.74	1.1	2.07	1.99	2.53	2.71	4.93	7.52
	244	5.19	4.23	1.91	2.75	3.63	2.92	1	3.44	3.14	2.71	2.01	2.4	3.67	0.77	0.32	2.8	2.27	1.02	2.6	2.99	3.82	3.63	3.14	1.82	1.29	0.72	0	0.77	2.4	1.68	1.87	1.07	1.62	1.35	1.39	2.17	2.53	4.91	7.53
	207	4.56	3.59	1.32	2.11	3.06	2.72	0.82	3.4	3.33	2.76	1.99	2.63	3.95	0.73	0.57	3.33	2.72	1.49	2.99	3.63	4.46	4.21	3.64	2.57	2.05	1.49	0.77	0	2.83	2.16	2.52	1.73	2.31	0.58	1.04	2.08	2.62	5.08	7.68
Galata Köprüsü	228	7.34	6.37	4.14	4.92	5.88	5.32	3.4	5.78	5.31	5.02	4.38	4.57	5.68	3.17	2.71	4.39	4.17	3.17	4.54	4.2	4.86	4.93	4.79	1.75	2.32	2.24	2.4	2.83	0	0.73	1.1	1.51	1.4	3.23	2.31	1.7	1.2	2.74	5.3
	173	6.71	5.74	3.47	4.27	5.21	4.6	2.68	5.06	4.6	4.29	3.65	3.86	5	2.45	1.99	3.77	3.49	2.45	3.86	3.65	4.36	4.38	4.17	1.31	1.69	1.53	1.68	2.16	0.73	0	0.75	0.8	0.89	2.61	1.82	1.58	1.39	3.4	5.99
	225	7.05	6.09	3.78	4.6	5.5	4.64	2.85	4.97	4.38	4.19	3.64	3.67	4.68	2.61	2.19	3.33	3.18	2.35	3.55	3.1	3.75	3.83	3.73	0.65	1.31	1.42	1.87	2.52	1.1	0.75	0	0.8	0.35	3.05	2.44	2.32	2.09	3.82	6.33
	62	6.25	5.29	2.97	3.8	4.7	3.89	2.05	4.3	3.8	3.52	2.92	3.07	4.2	1.82	1.39	3	2.69	1.67	3.06	2.94	3.69	3.65	3.39	0.92	0.94	0.74	1.07	1.73	1.51	0.8	0.8	0	0.59	2.28	1.82	2.03	2.07	4.2	6.79
	61	6.8	5.85	3.53	4.36	5.23	4.32	2.56	4.63	4.03	3.85	3.31	3.32	4.34	2.33	1.93	3	2.83	2.01	3.21	2.8	3.49	3.54	3.4	0.42	0.96	1.1	1.62	2.31	1.4	0.89	0.35	0.59	0	2.87	2.37	2.41	2.28	4.14	6.66
Avrasya Tüneli	175	4.11	3.14	1.03	1.71	2.69	2.72	1.11	3.5	3.59	2.94	2.18	2.94	4.26	1.13	1.14	3.8	3.15	1.99	3.38	4.15	4.97	4.69	4.08	3.14	2.64	2.07	1.35	0.58	3.23	2.61	3.05	2.28	2.87	0	1.09	2.18	2.82	5.26	7.83
	270	5.11	4.14	2.12	2.77	3.76	3.72	1.86	4.44	4.36	3.79	3.03	3.64	4.95	1.74	1.43	4.19	3.64	2.38	3.94	4.38	5.19	5.02	4.53	2.73	2.52	1.99	1.39	1.04	2.31	1.82	2.44	1.82	2.37	1.09	0	1.1	1.74	4.17	6.75
	230	6.11	5.16	3.21	3.83	4.83	4.8	2.88	5.47	5.29	4.79	4.04	4.55	5.84	2.72	2.33	4.88	4.42	3.19	4.76	4.93	5.71	5.63	5.25	2.83	2.94	2.53	2.17	2.08	1.7	1.58	2.32	2.03	2.41	2.18	1.1	0	0.72	3.09	5.65
	282	6.81	5.86	3.84	4.5	5.5	5.33	3.37	5.94	5.66	5.23	4.5	4.91	6.15	3.19	2.75	5.05	4.68	3.5	5.04	5	5.74	5.72	5.44	2.7	3.01	2.71	2.53	2.62	1.2	1.39	2.09	2.07	2.28	2.82	1.74	0.72	0	2.46	5.07
	82	9.14	8.21	6.29	6.92	7.91	7.78	5.81	8.36	7.99	7.62	6.92	7.24	8.4	5.62	5.17	7.14	6.89	5.82	7.27	6.92	7.53	7.65	7.54	4.46	5.06	4.93	4.91	5.08	2.74	3.4	3.82	4.2	4.14	5.26	4.17	3.09	2.46	0	2.62
	84	11.58	10.68	8.87	9.45	10.44	10.39	8.43	10.97	10.59	10.23	9.54	9.84	10.98	8.24	7.79	9.66	9.46	8.42	9.84	9.38	9.92	10.1	10.06	6.94	7.61	7.52	7.53	7.68	5.3	5.99	6.33	6.79	6.66	7.83	6.75	5.65	5.07	2.62	0

15.04.2023 24 saat süresince OD çiftleri arasındaki seyahat süreleri.

**Table 3:** Bluetooth sensor data organizing.

Time	Idaut	Start Time	End Time	Duration Time	Idsen
1.04.2023 00:00	20516747	1.04.2023 00:49	1.04.2023 00:49	0 days 00:00:00	[229]
1.04.2023 00:00	20616156	1.04.2023 00:51	1.04.2023 00:59	0 days 00:08:09	[254 177 248]
1.04.2023 00:00	20890057	1.04.2023 00:49	1.04.2023 00:49	0 days 00:00:00	[271]
1.04.2023 00:00	20911081	1.04.2023 00:31	1.04.2023 00:32	0 days 00:01:10	[170 169]
1.04.2023 00:00	20911365	1.04.2023 00:35	1.04.2023 00:59	0 days 00:24:07	[153]
1.04.2023 00:00	20947841	1.04.2023 00:49	1.04.2023 00:55	0 days 00:05:50	[171 169]
1.04.2023 00:00	21095198	1.04.2023 00:43	1.04.2023 00:43	0 days 00:00:00	[171 113 170 174]
1.04.2023 00:00	21098109	1.04.2023 00:51	1.04.2023 00:51	0 days 00:00:06	[115 169]
1.04.2023 00:00	21264328	1.04.2023 00:57	1.04.2023 00:57	0 days 00:00:06	[226]
1.04.2023 00:00	21378177	1.04.2023 00:30	1.04.2023 00:37	0 days 00:06:08	[254 244]
1.04.2023 00:00	21628717	1.04.2023 00:41	1.04.2023 00:41	0 days 00:00:00	[226]
1.04.2023 00:00	21758057	1.04.2023 00:49	1.04.2023 00:53	0 days 00:04:03	[227 241 226]
1.04.2023 00:00	22014812	1.04.2023 00:30	1.04.2023 00:30	0 days 00:00:00	[115]
1.04.2023 00:00	22019794	1.04.2023 00:29	1.04.2023 00:45	0 days 00:16:38	[227 174 172]
1.04.2023 00:00	22039229	1.04.2023 00:57	1.04.2023 00:57	0 days 00:00:00	[226]
1.04.2023 00:00	22202826	1.04.2023 00:29	1.04.2023 00:29	0 days 00:00:44	[241]

**Table 4:** Example section of speed values of Bluetooth devices.

Idaut	Idsen	Start Time	End Time	Speed (km/sa)
2369641	271	1.04.2023 07:01	1.04.2023 07:01	12.179
95977528	244	3.04.2023 23:55	3.04.2023 23:56	12.179
10019743	173	6.04.2023 04:13	6.04.2023 04:13	12.179
1924159	169	7.04.2023 18:50	7.04.2023 18:50	12.179
32926331	169	9.04.2023 18:50	9.04.2023 18:50	12.179
2947560	169	9.04.2023 19:20	9.04.2023 19:20	12.179
12129505	225	15.04.2023 09:23	15.04.2023 09:23	51.879
191503986	244	15.04.2023 09:23	15.04.2023 09:23	51.879
4128830	244	8.04.2023 14:41	8.04.2023 14:42	51.879
52223326	61	9.04.2023 08:16	9.04.2023 08:16	51.879
1398611	170	9.04.2023 08:32	9.04.2023 08:32	51.879
398057	244	9.04.2023 08:16	9.04.2023 08:16	51.879
23782274	227	15.04.2023 09:22	15.04.2023 09:23	51.879
321497324	194	15.04.2023 09:22	15.04.2023 09:23	80.001
1798186	225	10.04.2023 03:35	10.04.2023 03:36	93.601
34918	230	9.04.2023 13:09	9.04.2023 13:09	100.754
22249118	225	10.04.2023 03:35	10.04.2023 03:36	101.097
226920	82	3.04.2023 23:05	3.04.2023 23:05	101.097
33442150	230	7.04.2023 05:47	7.04.2023 05:47	101.097
33844008	230	8.04.2023 07:19	8.04.2023 07:19	101.097
758769	82	8.04.2023 11:50	8.04.2023 11:50	101.097
5609040	225	9.04.2023 23:05	9.04.2023 23:05	101.097
951487	153	16.04.2023 02:42	16.04.2023 02:46	101.097
268531398	225	3.04.2023 23:35	3.04.2023 23:37	19.211

**Table 5:** Sample section including Bluetooth IDs separated by vehicles.

Time	Idaut	Start Time	End Time	Duration Time	Idsen
2.04.2023 07:00	2250192	2.04.2023 07:27	2.04.2023 07:41	0 days 00:14:30	[61 113 248 271 174]
4.04.2023 03:00	2433122	4.04.2023 03:29	4.04.2023 03:39	0 days 00:10:00	[61 187 241 169]
9.04.2023 12:00	6042	9.04.2023 12:26	9.04.2023 12:39	0 days 00:13:29	[113 248 207 271 174]
8.04.2023 06:00	579297	8.04.2023 06:42	8.04.2023 06:39	0 days 00:03:03	[170 277 174 172 229]
10.04.2023 01:00	4091924	10.04.2023 01:10	10.04.2023 01:57	0 days 00:47:04	[153 113 171 241 170]
6.04.2023 20:00	2591564	6.04.2023 20:45	6.04.2023 20:57	0 days 00:12:06	[173 248 174 170]
9.04.2023 21:00	6514961	9.04.2023 21:44	9.04.2023 21:59	0 days 00:15:29	[173 248 174 170]
13.04.2023 23:00	352306125	13.04.2023 23:00	13.04.2023 23:32	0 days 00:31:48	[173 248 174 170]
6.04.2023 23:00	613126	6.04.2023 23:45	6.04.2023 23:57	0 days 00:12:15	[225 187 169]
9.04.2023 21:00	44362575	9.04.2023 21:05	9.04.2023 21:16	0 days 00:11:30	[226 241 227 175 207]
13.04.2023 01:00	209199330	13.04.2023 01:20	13.04.2023 01:49	0 days 00:29:14	[230 226 241 207 172]
6.04.2023 01:00	273117	6.04.2023 01:09	6.04.2023 01:37	0 days 00:28:00	[244 113 271 170]
6.04.2023 23:00	560193	6.04.2023 23:28	6.04.2023 23:33	0 days 00:05:35	[170 277 172 229 172]
1.04.2023 13:00	538351	1.04.2023 13:53	1.04.2023 13:59	0 days 00:05:11	[275 272 230]
2.04.2023 01:00	112797035	2.04.2023 01:09	2.04.2023 01:15	0 days 00:06:27	[275 272 230]

**Table 6:** Speed-based filtering distribution.

Speed-Based Filtering			
Personal Devices ID	Pedestrian ID	3,042,551	57.12%
	P.T. ID	42,394	6.99%
	L.R. ID	42,747	7.45%
Vehicle ID		217,348	35.82%

**Table 7:** Jaccard index.

	Time-Based Filtering	Speed-Based Filtering
Vehicle ID	208,941	217,348
Vehicle ID (%)	34.43%	35.82%
Personal Devices ID	397,799	346,645
Personal Devices ID (%)	65.57%	64.17%
Jaccard Index (J)	0.628	
